# Impact of the COVID-19 pandemic on extended-spectrum β-lactamase producing *Escherichia coli* in urinary tract and blood stream infections: results from a nationwide surveillance network, Finland, 2018 to 2022

**DOI:** 10.1186/s13756-024-01427-z

**Published:** 2024-07-06

**Authors:** Heikki Ilmavirta, Jukka Ollgren, Kati Räisänen, Tuure Kinnunen, Antti Juhani Hakanen, Kaisu Rantakokko-Jalava, Jari Jalava, Outi Lyytikäinen

**Affiliations:** 1 Department of Clinical Microbiology, Institute of Clinical Medicine, University of Eastern Finland, Kuopio, Finland; 2 ISLAB Laboratory Centre, Kuopio, Finland; 3 Department of Health Security, Finnish Institute for Health and Welfare (THL), Helsinki, Finland; 4 Tyks Laboratories, Turku University Hospital (TYKS) and University of Turku (UTU), Turku, Finland

**Keywords:** Escherichia coli, ESBL, COVID-19, AMR, Urinary tract infection, Bloodstream infection, Male, Female, Finland, Decreased resistance

## Abstract

**Background:**

Before the COVID-19 pandemic there has been a constant increase in antimicrobial resistance (AMR) of *Escherichia coli*, the most common cause of urinary tract infections and bloodstream infections. The aim of this study was to investigate the impact of the COVID-19 pandemic on extended-spectrum β-lactamase (ESBL) production in urine and blood *E. coli* isolates in Finland to improve our understanding on the source attribution of this major multidrug-resistant pathogen.

**Methods:**

Susceptibility test results of 564,233 urine (88.3% from females) and 23,860 blood *E. coli* isolates (58.8% from females) were obtained from the nationwide surveillance database of Finnish clinical microbiology laboratories. Susceptibility testing was performed according to EUCAST guidelines. We compared ESBL-producing *E. coli* proportions and incidence before (2018–2019), during (2020–2021), and after (2022) the pandemic and stratified these by age groups and sex.

**Results:**

The annual number of urine *E. coli* isolates tested for antimicrobial susceptibility decreased 23.3% during 2018–2022 whereas the number of blood *E. coli* isolates increased 1.1%. The annual proportion of ESBL-producing *E. coli* in urine *E. coli* isolates decreased 28.7% among males, from 6.9% (average during 2018–2019) to 4.9% in 2022, and 28.7% among females, from 3.0 to 2.1%. In blood *E. coli* isolates, the proportion decreased 32.9% among males, from 9.3 to 6.2%, and 26.6% among females, from 6.2 to 4.6%. A significant decreasing trend was also observed in most age groups, but risk remained highest among persons aged ≥ 60 years.

**Conclusions:**

The reduction in the proportions of ESBL-producing *E. coli* was comprehensive, covering both specimen types, both sexes, and all age groups, showing that the continuously increasing trends could be reversed. Decrease in international travel and antimicrobial use were likely behind this reduction, suggesting that informing travellers about the risk of multidrug-resistant bacteria, hygiene measures, and appropriate antimicrobial use is crucial in prevention. Evaluation of infection control measures in healthcare settings could be beneficial, especially in long-term care.

**Supplementary Information:**

The online version contains supplementary material available at 10.1186/s13756-024-01427-z.

## Background

Antimicrobial resistance (AMR) has emerged as one of the leading public health threats in the 21st century [1, 2]. AMR is accelerated by misuse or overuse of antimicrobials and poor infection prevention and control (IPC) [3]. Hence, antimicrobial stewardship programs and IPC have been used as mitigation strategies against AMR. In addition, several other factors may contribute to AMR, such as the presence of multidrug-resistant (MDR) bacteria in livestock and agricultural products, and increasing foreign travel [4], particularly to countries with a high prevalence of MDR bacteria. The onset of the COVID-19 pandemic affected healthcare systems, causing major disruptions that threaten the effectiveness of IPC and antimicrobial stewardship strategies [5, 6]. The COVID-19 pandemic also complicated AMR surveillance and research, as changes in healthcare delivery, improved IPC measures related to the pandemic, and reduced national and international travel may have reduced the selection of pathogens resistant to antimicrobials in a short term [7]. However, opposite impacts could also be seen if antimicrobials have been used more frequently and inappropriately during the pandemic.

*Escherichia coli* is the leading cause of urinary tract infections (UTI) and bloodstream infections (BSI) worldwide, causing substantial and increasing burden of disease, especially among elderly people [8–12]. The emergence of AMR among *E. coli* causes major concern, as infections caused by MDR *E. coli* are more challenging to treat, conferring a higher risk of bacteraemia and death [13]. Extended-spectrum β-lactamase (ESBL) production provides resistance to many clinically important antimicrobials, including third-generation cephalosporins (3GC), which are widely used as the first-line empirical treatment in severe *E. coli* infections, such as pyelonephritis or BSI. Several recent surveillance reports have demonstrated a decrease in the proportion of ESBL-producing or 3GC-resistant *E. coli* during the pandemic years 2020–2022 [14–17]. Also, in the latest report of European Antimicrobial Resistance Surveillance Network (EARS-Net), there was an overall decreasing trend of 3GC-resistance in invasive *E. coli* isolates [18]. However, these surveillance reports have rarely covered both urine and blood isolates or evaluated the proportions and risk in different age groups and sex.

Our previous study covering the years preceding the COVID-19 pandemic (2008–2019) demonstrated an average annual increase (AAI) of around 9% in the proportion of ESBL-producing *E. coli* among urine and blood *E. coli* isolates in Finland, and this increase was similar in all age groups regardless of sex [19]. In the current study, we investigated the impact of the COVID-19 pandemic on the epidemiology of ESBL-producing *E. coli* and analysed the trends in the proportion of ESBL-producing isolates among *E. coli* isolated from blood or urine cultures in different age groups and both sexes during and after the COVID-19 pandemic. We also assessed the changes in the incidence of ESBL-producing *E. coli* during the study period.

## Methods

The national Finres database [20] contains antimicrobial susceptibility test results of 20 common clinically important bacteria under surveillance in Finland, including *E. coli* [19]. For each bacterial species, only the first isolate with a susceptibility test result per sample type and patient is reported to this database annually. Information collected includes bacterial name, susceptibility test results for selected antimicrobials (disc diffusion, minimum inhibitory concentration, interpretation of the test result, and/or confirmed resistance mechanism), age and sex (male or female), and date and type of specimen. Antimicrobial susceptibility tests including phenotypical ESBL screening and confirmation were performed and interpreted according to the European Committee on Antimicrobial Susceptibility Testing (EUCAST) guidelines [21]. The described data are reported annually by all Finnish clinical microbiology laboratories, covering all healthcare districts in Finland. All laboratories are government licenced and participate in international and national external quality assessment programmes including the EARS-Net quality control scheme. During 2018–2022, the annual number of laboratories reporting blood cultures varied between 15 and 19 and the number of those reporting urine cultures between 15 and 21. The Finres database covered 95% (range by year 87–100%) of all blood [22] and approximately 90% of all urine culture isolates sampled in Finland during the study period.

### Analysis and statistics

To minimize bias, we excluded one laboratory accounting annually an average of 9.9% of all blood and 7.8% of all urine isolates in the Finres database during 2018–2021, since the laboratory was not able to report susceptibility test results for 2022 due to technical reasons.

We calculated the annual proportions of ESBL-producing *E. coli* isolates from all urine and blood *E. coli i*solates for different sexes and age groups, and the annual proportion of fluoroquinolone resistant ESBL-producing *E. coli* isolates, defined as ESBL-producing and resistant to moxifloxacin, levofloxacin, ciprofloxacin, and/or norfloxacin. We also calculated the annual incidences of ESBL-producing *E. coli* per 100,000 inhabitants. To compare observed trends over time and between age groups and sex, we applied a binomial regression model with log link and with or without Newey–West standard errors, which take into account the possible autocorrelation conditional on the chosen trend. For average annual decreases (AAD) and trends, we calculated 95% compatibility intervals (CI) and p values, p values of < 0.05 were considered statistically significant. In addition, we calculated the mean annual proportion of ESBL-producing *E. coli* isolates during 2018–2019 and compared it to the proportion in 2022 to assess the relative and absolute change during the pandemic. Data were analysed using SPSS Statistics 25 (IBM, .ibm.com) and Stata 17.0 (StataCorp LLC, .stata.com).

## Results

During 2018–2022, a total of 848,168 urine culture and 56,788 blood culture bacterial isolates were identified in the Finres database; 93.4% (792,526/848,168) of the urine isolates and 92.0% (52,219/56,788) of the blood isolates were included in our analyses. Of the included isolates, 71.2% (564,233/792,526) of the urine isolates and 45.7% (23,860/52,219) of the blood isolates were identified as *E. coli*.

The total annual number of all urine isolates decreased by 22.5% during the study period, from 176,904 in 2018 to 137,013 in 2022, but remained stable for blood isolates (9,970 in 2018, 10,947 in 2019, and 10,662 in 2022). Similarly, the total annual number of urine *E. coli* isolates tested for antimicrobial susceptibility decreased by 23.3%, from 125,315 in 2018 to 96,123 in 2022, whereas the number of blood isolates increased by 1.1%, from 4,523 in 2018 to 4575 in 2022. Of urine *E. coli* isolates, 88.3% (498,162/564,233) were from females and 11.7% (66,071/564,233) from males, and 58.8% (14,020/23,860) of blood *E. coli* isolates were from females and 41.2% (9,840/23,860) from males. The proportions of urine and blood *E. coli* isolates from patients aged ≥ 60 years were 71.1% (401,044/564,233) and 84.5% (20,170/23,860), respectively.

For all *E. coli* isolates, information of their ESBL status was available. In addition, susceptibility test result for at least one fluoroquinolone was available for 98.8% (557,518/564,233) of the urine and 99.8% (23,809/23,860) of the blood *E. coli* isolates.

**Fig. 1:**
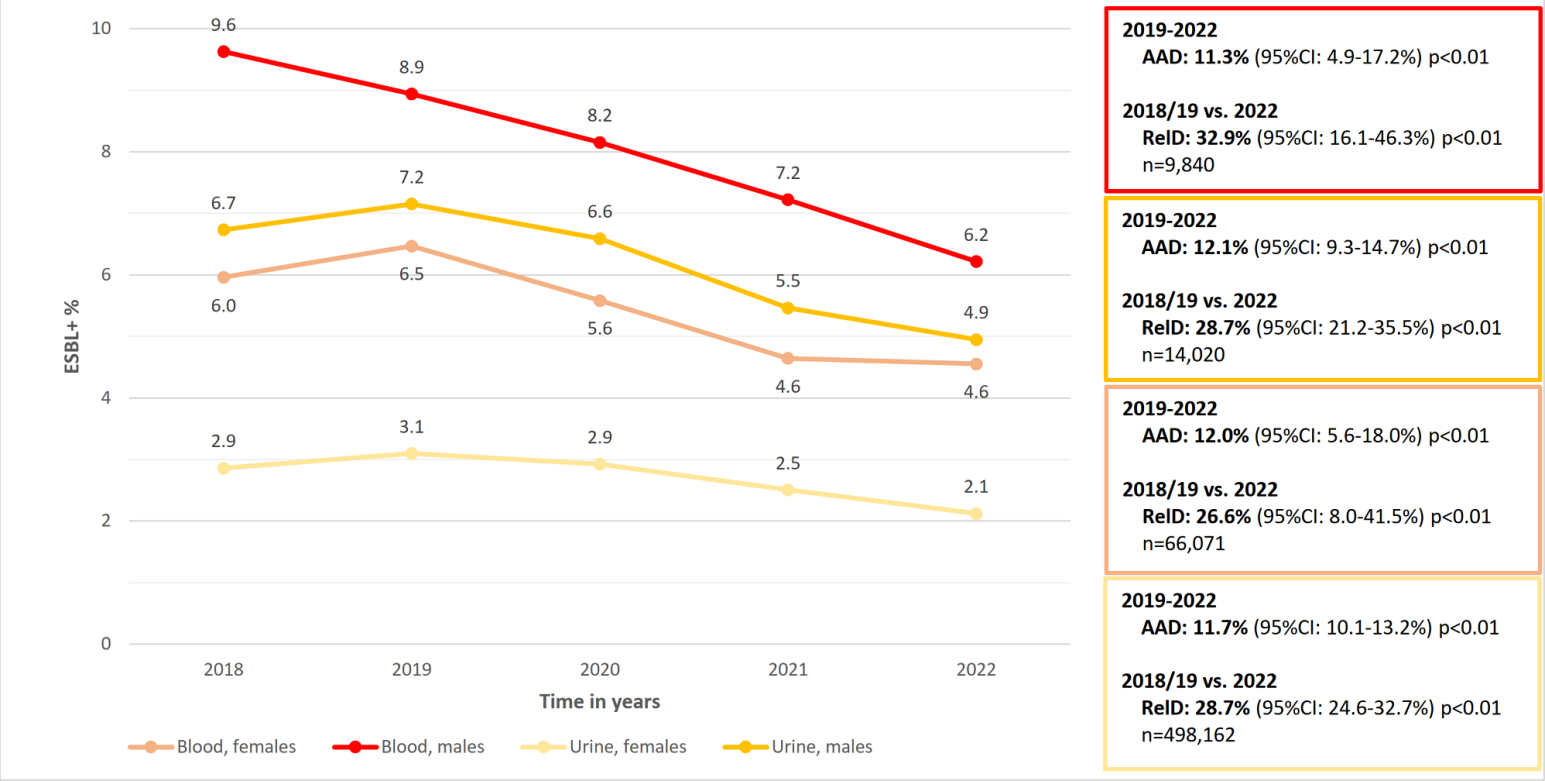
The annual proportion of extended-spectrum β-lactamase-producing *Escherichia coli* in blood and urine *E. coli* isolates among males and females, Finland, 2018–2022. AAD: average annual decrease; CI: compatibility interval; ESBL+: extended-spectrum β-lactamase-producing *Escherichia coli*; RelD: relative decrease

During 2019–2022, a significant decreasing trend in the annual proportion of ESBL-producing *E. coli* in urine and blood *E. coli* isolates was observed in both males and females (Fig. 1 and Supplementary Table S1). In urine isolates, the decrease averaged 27.6% among both sexes from 2018/19 to 2022: among males from 6.9 to 4.9% (AAD: 12.1%, 95%CI: 9.3–14.7%, *p* < 0.01) and among females from 3.0 to 2.1% (AAD: 11.7%, 95%CI: 10.1–13.2%, *p* < 0.01). In blood isolates, the proportion decreased by an average of 29.0% from 2018/19 to 2022: among males from 9.3 to 6.2% (AAD: 11.3%, 95%CI: 4.9–17.2%, *p* < 0.01) and among females from 6.2 to 4.6% (AAD: 12.0%, 95%CI: 5.6–18.0%, *p* < 0.01). The AAD values were similar for both urine and blood isolates for both sexes during the study period. Notably, the annual proportion of ESBL-producing *E. coli* was constantly higher in blood than in urine *E. coli* isolates and higher in males than in females.

**Fig. 2:**
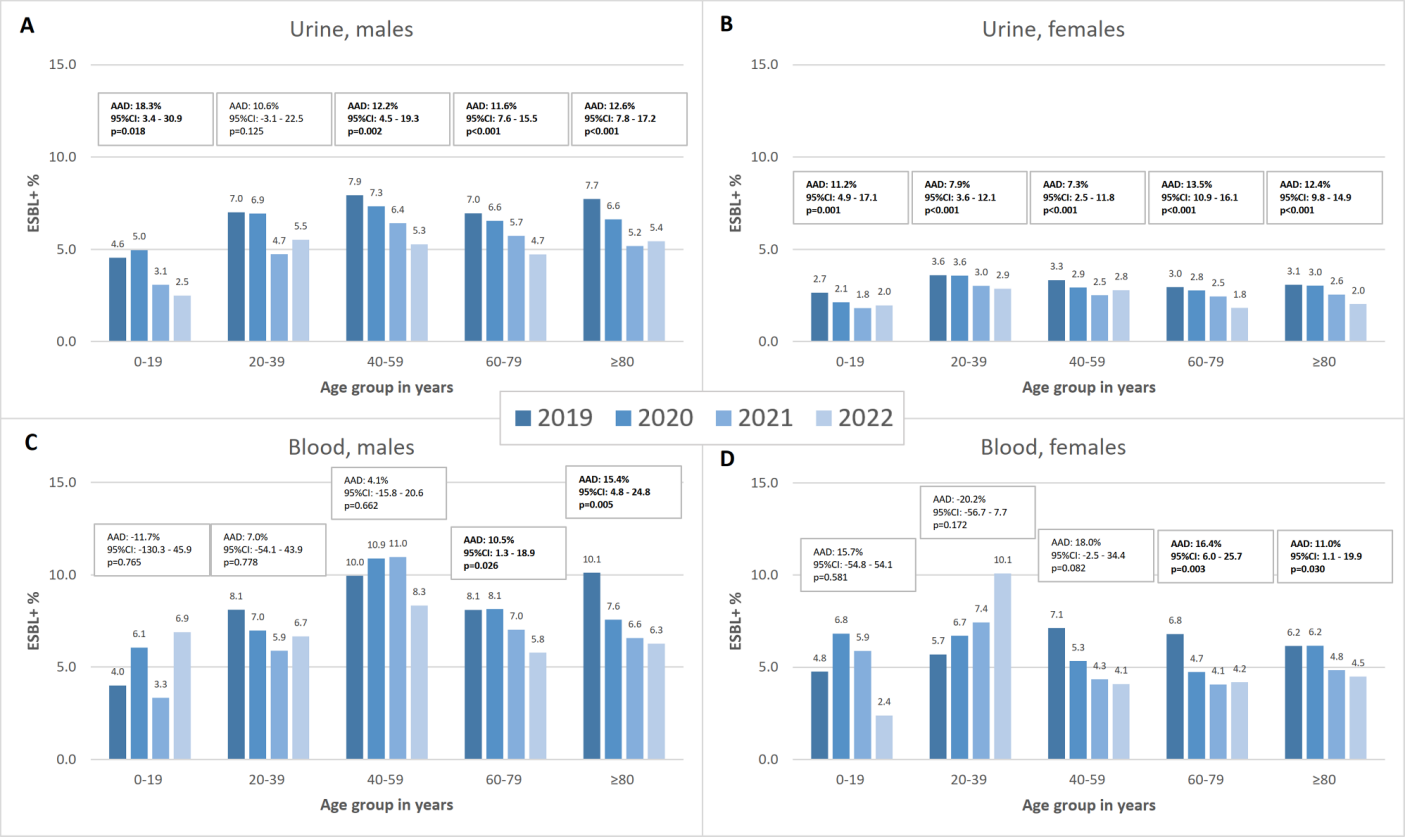
The annual proportion of extended-spectrum β-lactamase-producing *Escherichia coli* in urine *E. coli* isolates among (**A**) males and (**B**) females and in blood *E. coli* isolates among (**C**) males and (**D**) females, Finland, 2019–2022. AAD: average annual decrease; CI: compatibility interval; ESBL+: extended-spectrum β-lactamase-producing *Escherichia coli*

Importantly, the annual proportion of ESBL-producing *E. coli* in urine and blood *E. coli* isolates decreased in all age groups during 2019–2022, except for blood isolates from males aged 0–19 years and females aged 20–39 years (Fig. 2 and Supplementary Table S1). In urine isolates, the decreasing trend was statistically significant in all age groups, except for males aged 20–39 years, but in blood isolates significant only in age groups of 60–79 and ≥ 80 years among both sexes. When considering the 95% CIs, the significant trends (AADs) were very similar in different age groups for both sexes.

**Fig. 3:**
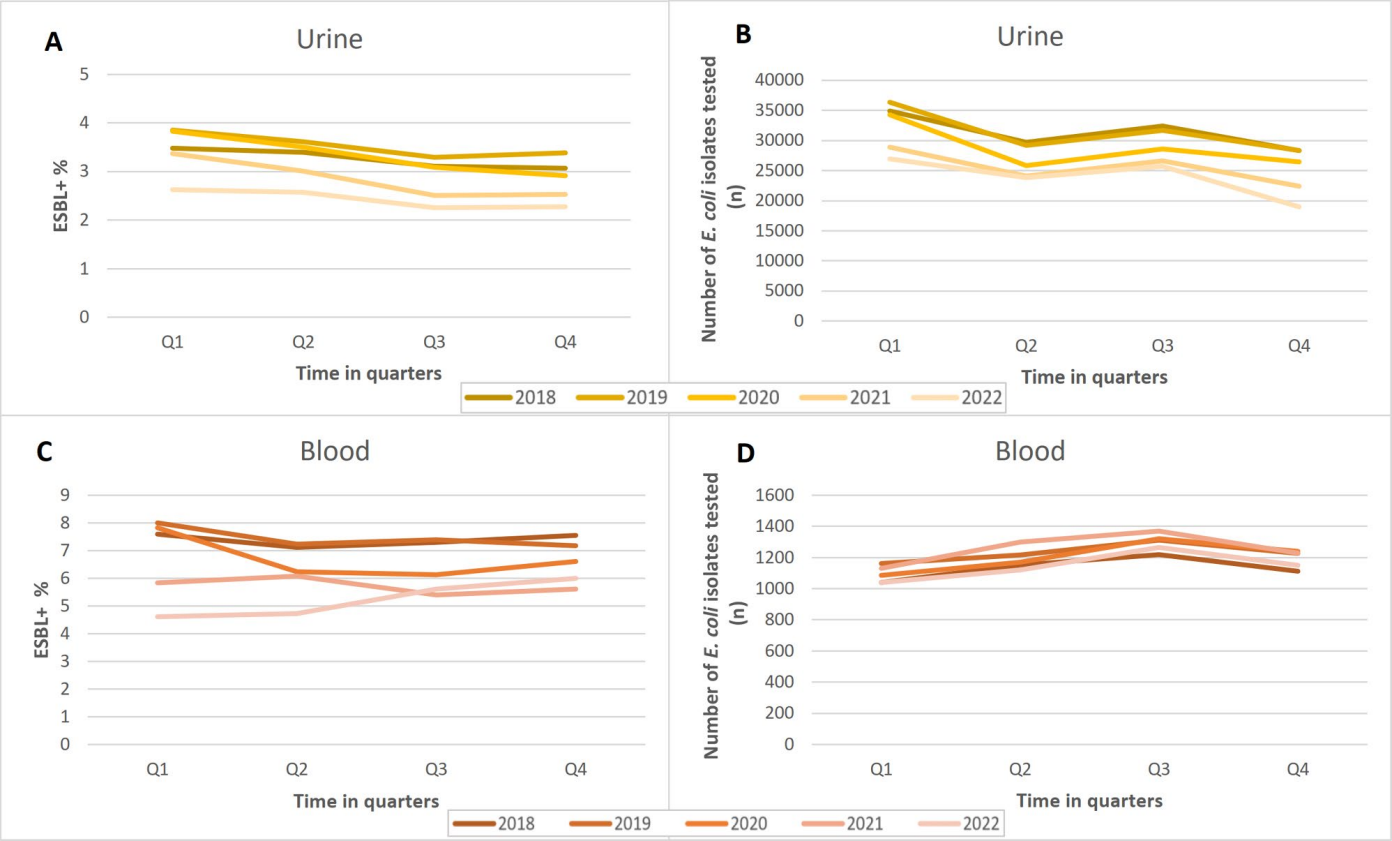
The quarterly analysis of the proportions of extended-spectrum β-lactamase-producing *Escherichia coli* in (**A**) urine and (**C**) blood *E. coli* isolates and the number of urine (**B**) and blood (**D**) *E. coli* isolates tested, Finland, 2018–2022. ESBL+: extended-spectrum β-lactamase-producing *Escherichia coli*, Q1-Q4: quarter 1–4

Quarterly analysis shows that, the proportion of ESBL-producing *E. coli* isolates started to decrease during quarter 2 and 3 of 2020 for urine and during quarter 2 of 2020 for blood isolates – immediately after the onset of the pandemic (quarter 1 of 2020) (Fig. 3A and C). In quarter 3 and 4 of 2022, this decrease stabilized for urine isolates and started to increase again for blood isolates. Although the numbers of urine *E. coli* isolates tested decreased during the pandemic years, quarterly testing activity remained rather unchanged throughout the study period (Fig. 3B).

**Fig. 4:**
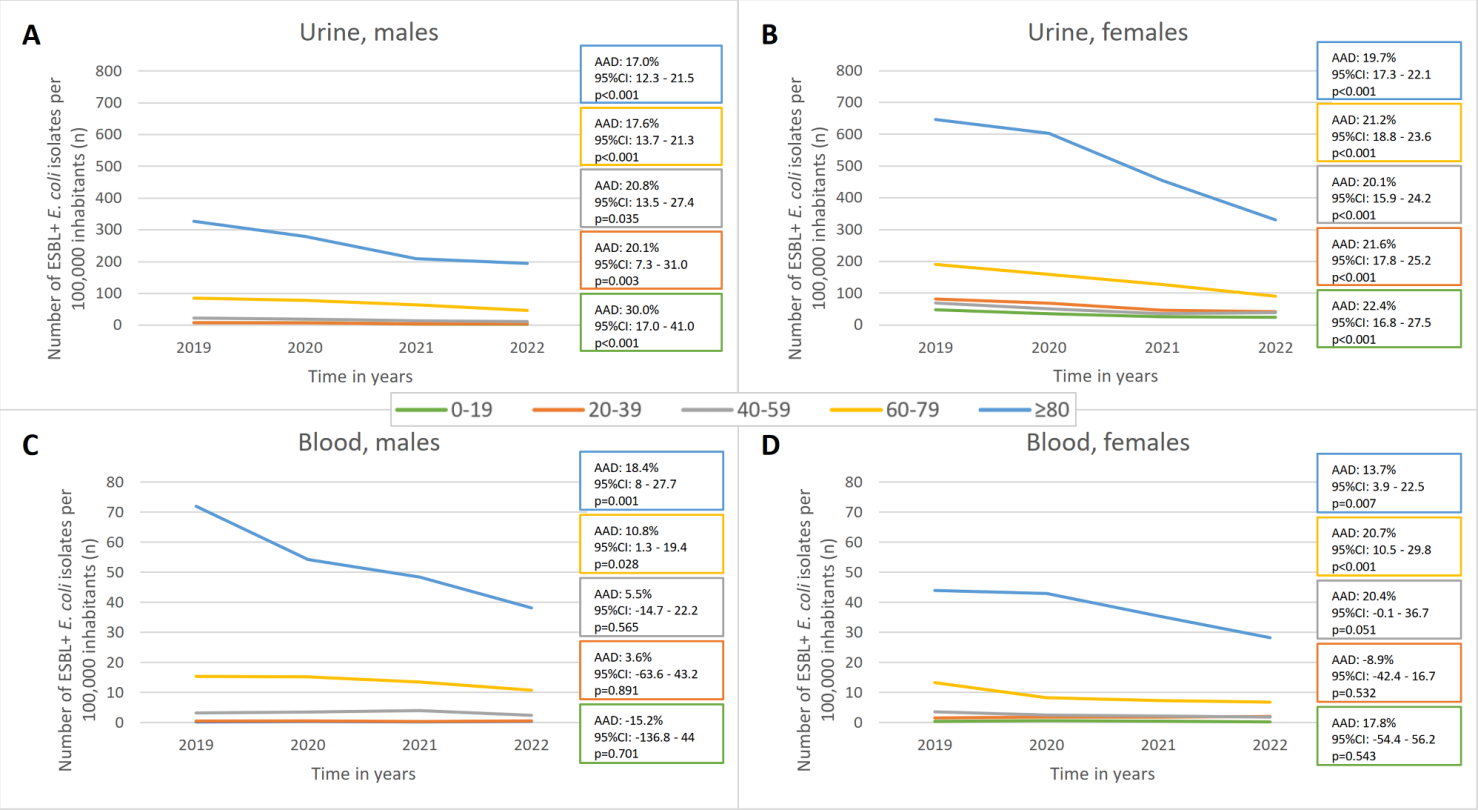
The annual incidence of extended-spectrum β-lactamase-producing *Escherichia coli* (numbers per 100,000 inhabitants) in urine cultures among (**A**) males and (**B**) females and in blood cultures among (**C**) males and (**D**) females, Finland, 2019–2022. AAD: average annual decrease; CI: compatibility interval; ESBL+: extended-spectrum β-lactamase-producing *Escherichia coli*

The incidence of ESBL-producing *E. coli* in urine and blood cultures decreased in most age groups during 2019–2022 among both sexes (Fig. 4 and Supplementary Table S2). However, the incidence decreased overall more in urine isolates than in blood isolates. In urine cultures, the decrease averaged 44.8% from 2018/19 to 2022: among males from 38.6 per 100.000 inhabitants in 2018/19 to 23.6 in 2022 (AAD: 17.2%, 95%CI: 14.5–19.7%, *p* < 0.01) and among females from 131.3 to 70.3 (AAD: 20.0%, 95%CI: 18.6–21.4%, *p* < 0.01). In blood cultures, the incidence decreased by an average of 31.6% from 2018/19 to 2022: among males from 7.0 to 4.9 (AAD: 11.1%, 95%CI: 4.5–17.2%, *p* < 0.01) and among females from 7.0 to 4.7 (AAD: 14.6%, 95%CI: 8.2–20.6%, *p* < 0.01). This decreasing trend was significant in all age groups for urine isolates, but for blood isolates only in males and females aged ≥ 60 years. When considering the 95% CIs, the significant trends (AADs) were very similar in different age groups for both sexes. The largest decrease in the incidence was observed in the two oldest age groups (60–79 and ≥ 80 years), being particularly prominent in persons aged ≥ 80 years.

The proportion of fluoroquinolone-resistant isolates among ESBL-producing *E. coli* decreased only slightly during 2019–2022: in urine isolates from 67.6 to 64.5% and in blood isolates from 73.1 to 66.4%. Of note, *E. coli* isolates resistant (R) or susceptible with increased exposure (I) to meropenem or imipenem were rare during the study period: a total of 28 isolates in urine (range by year, 2–8; 0.014% (28/200,605)) and 5 isolates in blood (range by year, 0–2; 0.019% (5/25,855)).

## Discussion

Our study based on the national surveillance data indicates that the annual proportions of ESBL-producing isolates among *E. coli* from urine and blood cultures significantly decreased after the onset of the COVID-19 pandemic during 2019–2022. Concurrently, the incidence of ESBL-producing *E. coli* significantly decreased in urine cultures in both sexes in all age groups, and also in blood cultures of both males and females ≥ 60 years of age. In addition, we observed a clear decrease in the annual number of urine isolates reported to the surveillance database during the pandemic. However, for blood isolates, there was a slight increase during this timeframe. Furthermore, for ESBL-producing *E. coli* isolates, coincident resistance to fluoroquinolones remained high during the study period.

Our study shows that, the observed decreasing trends in the proportion of ESBL-producing *E. coli* were more than a mirror image of the trends observed in our previous study in Finland covering the pre-pandemic years 2008–2019 (AAD during 2019–2022: 11.3% in urine and 11.4% in blood (Supplementary table S2) vs. AAI during 2008–2019: 8.9% in urine and 8.7% in blood) [19]. The lowest proportions observed in this study in 2022 were roughly at the same level as observed in 2015, 5 years before the onset of the pandemic. Notably, the previously observed differences between sexes and sample types in the levels of the proportions remained the same, with the proportion of ESBL-producing isolates being higher among males than females and higher in blood isolates than in urine isolates. Our results are also partly paralleled by three previous studies [23–25]. In France, an overall significant decrease in ESBL production among *E. coli* isolates from clinical samples of primary care patients and nursing home residents was reported after the national lockdown on the 11th of May 2020 [23]. The decrease was statistically significant for urine cultures, females, and the age groups of 5–19, 40–64, and > 60 years. In Ontario, Canada, a decreasing trend for ESBL-producing *E. coli* in urine cultures from community patients and patients in long-term care facilities (LTCF) during the COVID-19 pandemic was also observed [24]. However, the study periods in these two studies were shorter than ours. In the Netherlands, in hospitalised patients, a significantly lower prevalence of ESBL-producing *E. coli* and *Klebsiella pneumoniae* was observed from June to August 2022 compared to the pre-COVID-19 period [25]. In addition to these studies, a review including 30 studies demonstrated differences in trends of different MDR bacteria during the pandemic [26]: the proportions of ESBL-producing *E. coli* and *K. pneumoniae* and carbapenem-resistant *Pseudomonas aeruginosa* (CRPA) decreased in most studies, whereas the proportions of other MDR bacteria including carbapenem-resistant *Enterobacteriaceae* (CRE), carbapenem-resistant *Acinetobacter baumannii* (CRAB), methicillin-resistant *Staphylococcus aureus* (MRSA), and vancomycin-resistant *Enterococci* (VRE) increased.

The decreasing trends in the proportions of ESBL-producing *E. coli* in blood *E. coli* isolates observed in this study are in line with the latest EARS-Net report for years 2018–2022, although EARS-Net reports 3GC-resistance proportion instead of ESBL proportion [18, 22]. The population-weighted mean proportion of 3GC resistance among invasive *E. coli* isolates decreased by 22.8%: from 7.9% in 2019 to 6.1% in 2022 in Finland [22]. This was nearly triple compared to the mean decrease in European Union (EU) and European Economic Area (EEA) countries (8.3%, from 15.6 to 14.3%), as well as greater than in some other European countries with traditionally low rates of AMR: Norway, 6.5%, from 6.2% in 2019 to 5.8% in 2022; Sweden, 3.8%, from 7.8 to 7.5%; and Denmark, 12,0%, from 7.5 to 6.6%. Moreover, in contrast to what we observed in Finland, the lowest 3CG resistance proportions were encountered already in 2021 in these countries, after which the trend may have reversed. Interestingly, in the Netherlands, the proportion of 3GC resistance remained stable during the COVID-19 pandemic (7.5% in 2019 and 7.7% in 2022). A similar phenomenon has also been reported in the UK, which was not included in the latest EARS-Net reports, where 3GC *E. coli* resistance in BSIs remained relatively stable at 14.5% between 2018 and 2022 [27]. Notably, in one Nordic country, Iceland, the proportion of 3GC resistance actually increased by 40%, from 7.0% in 2019 to 9.8% in 2022 [22].

For urine *E. coli* isolates, during 2019–2022, decreasing trends for cefadroxil-resistant isolates (representing ESBL-producing isolates) and 3GC-resistant isolates have been reported in the national surveillance reports of Sweden and Denmark, respectively [15, 17]. However, the relative changes in these proportions were again smaller than observed in our study in Finland. Moreover, the lowest proportions of these isolates were reported already in 2021, after which the trend may have reversed, contrasting the data from Finland (Sweden: from 6.2% in 2019 to 5.9% in 2021 and 6.2% in 2022; Denmark: at hospital level from 6.9 to 5.8% in 2021 and 6.2% in 2022 and at primary health care from 5.2 to 4.4% in 2021 and 4.8% in 2022). Of note, in Norway, the proportion of ESBL-producing isolates was not reported to decrease among urine *E. coli* isolates during 2019–2022 [16]. However, the proportion there remained very low (3.0% in 2019 and 3.8% in 2022).

In contrast to most previous studies and reports, we showed that, although the decreases in the proportions of ESBL-producing *E. coli* were similar in most demographic groups and between sample types, the decreases in the incidence differed, reflecting the changes in tested isolates during the study period. The observed decrease in the total annual number of urine isolates tested and the resulting decrease in the number of *E. coli* isolates might reflect changes in diagnostic activity of UTIs or healthcare service access after the onset of the pandemic. Hence, particularly uncomplicated and/or non-severe UTIs may have been underdiagnosed during the pandemic years. Due to this selection bias, the proportion of ESBL-producing *E. coli* among urine *E. coli* isolates may be slightly overestimated, and the actual annual decrease may have been even larger. In addition, the reduction in elective care in hospitals may have decreased routinely sampled urine cultures, further affecting the numbers and proportions. For blood isolates, the previously observed continuous increase in the annual numbers [19] nearly stopped. This raises a question whether BSIs were also underdiagnosed during the pandemic. Importantly, in both sample types, the annual testing patterns did not clearly change, and the proportion of *E. coli* as a causative agent of UTIs and BSIs remained similar to pre-pandemic period, 69.3% and 44.0% during 2008–2019 [19] and 71.0% and 46.1% during 2020–2022, respectively.

In the context of COVID-19 pandemic, several factors may have influenced the decreasing trends observed in this study [28, 29]. First, restrictions in travel, in particular international travel, may have significantly decreased the acquisition and cross-border import of ESBL-producing *E. coli* in Finland [30, 31]. The number of travellers in Finnish airports decreased dramatically from 1.9 million in February 2020 to less than half in March 2020 and to only 1% in April 2020 [32]. Thereafter, the annual number of travellers increased but was over 10 million less in 2022 (15.6 million) compared to the pre-pandemic year 2019 (26.3 million) [33]. Similar trends were seen in at Swedish, Danish, Norwegian, and Dutch airports [34–37]. The decreased import of ESBL-producing *E. coli* via travel likely leads also in reduced onward transmission within household members, which is known to occur in up to 12% of the cases [38]. Second, the selective pressure of antibacterials reduced during the study period. The total consumption of antibacterials for systemic use in Finland decreased by 14.9% from 2019 to 2022, which was the greatest decrease among EU countries during the pandemic (EU mean: -2,5%) [39]. In 2022, Finland was among the EU/EAA countries with the lowest antibacterial consumption. The decrease in antibacterial consumption has been related to more stringent hygiene measures in prevention of COVID-19, which also decreased the spread of other respiratory pathogens [40, 41] and resulted in the decreased usage of antibacterials. Third, the IPC measures in the hospitals and LTCFs in response to the pandemic may have decreased the spread of ESBL-producing *E. coli* in the health care setting [42].

Our study is not without limitations. First, the results of one major Finnish laboratory were not reported to the Finres database for year 2022. However, similar trends in the proportions of ESBL-producing isolates among blood *E. coli* isolates were observed according to local statistics (personal communication, KRJ, 13th of February 2024). Second, we do not know to what extent different factors (international travel, antimicrobial use, and IPC measures) contributed to the decrease in the proportions of ESBL-producing isolates. Third, the Finres database did not include information about community- or healthcare origin of the isolates. However, decreases in the proportions of ESBL-producing isolates in *E. coli* UTIs and BSIs in all age groups and both sexes suggest that the decrease likely happened in all settings, the community, acute care hospitals, and LTCFs. Last, we do not know whether the clinical outcome of these infections has changed during the study period.

## Conclusion

After the onset of the COVID-19 pandemic, the proportion of ESBL-producing *E. coli* in UTIs and BSIs caused by *E. coli* significantly decreased during 2019–2022. Simultaneously, the risk of these infections decreased in most age groups. Although decreasing trends were similar between most of the age groups, the decrease in risk was most conspicuous among people aged ≥ 60 years, particularly among those ≥ 80 years of age. Overall, our results suggests that the decrease likely happened concurrently in both the community and healthcare settings. We assume that the rapid and prominent decrease in international travel was a major contributing factor, accompanied by decreased antibiotic use and pandemic-related IPC measures. Therefore, informing travellers on the risk of MDR bacteria related to international travel, hygiene measures, and appropriate antimicrobial use is crucial and evaluation of infection control measures in healthcare settings could be beneficial, especially in long-term care. In quarters 3 and 4 of 2022, the decreasing trend in ESBL-producing *E. coli* appeared to stabilise in urine cultures and even started to increase in blood cultures. The future trends in these proportions in different sample types and demographic age groups may further inform about the causes of source attribution of ESBL-producing *E. coli*. Continuous monitoring of the situation is therefore necessary, and the factors contributing to this decrease require further investigation.

### Electronic supplementary material

Below is the link to the electronic supplementary material.

Supplementary Material 1

Supplementary Material 2

## Data Availability

The datasets analysed during the current study are mostly included in this published article and its supplementary material, and they are mostly available in the Finres database, https://sampo.thl.fi/pivot/prod/fi/finres/lite/fact_lite. Part of the data were used under license for the current study and so are not publicly available. These data are available from the Finnish Institute for Health and Welfare (THL) for reasonable request.

## Abbreviations

AAD: Average annual decrease
AAI: Average annual increase
AMR: Antimicrobial resistance
CI: Compatibility interval
CRAB: Carbapenem-resistant Acinetobacter baumannii
CRE: Carbapenem-resistant Enterobacteriaceae
CRPA: Carbapenem-resistant Pseudomonas aeruginosa
EAA: European Economic Area
EARS: Net - European Antimicrobial Resistance Surveillance Network
EU: European Union
EUCAST: European Committee on Antimicrobial Susceptibility Testing
ESBL: Extended-spectrum β-lactamase
IPC: Infection prevention and control
LTCF: Long-term care facility
MDR: Multidrug-resistant
MRSA: Methicillin-resistant Staphylococcus aureus
VRE: Vancomycin-resistant Enterococci
3CG: Third-generation cephalosporins
