# Supplementary material for: Impact of the COVID-19 pandemic on extended-spectrum β-lactamase producing Escherichia coli in urinary tract and blood stream infections: results from a nationwide surveillance network, Finland, 2018 to 2022
Source: Antimicrob Resist Infect Control. 2024 Jul 6;13:72. doi: 10.1186/s13756-024-01427-z (PMC11227720; doi:10.1186/s13756-024-01427-z)
Supplement: Supplementary file 1 — Supplementary Material 1 [file 13756_2024_1427_MOESM1_ESM.pdf]

Supplementary Table S1:

Supplementary Table S1: The annual proportions of ESBL-producing *Escherichia coli* in urine and blood *E. coli* isolates and their estimated relative decreaces stratified by age groups and both sexes, Finland, 2018-2022. AAD, Average annual decreace; ESBL+, extended-spectrum β-lactamase-producing *E. coli*; CI, combatibility interval.

Urine *E. coli*, MALE

Age group	Year	Observed resistance rate		2019-2022		2018/19 vs. 2022	
		ESBL+ (%)	Isolates tested (n)	p	AAD, % (95% CI)	p	Relative decrease, % (95% CI)
	2018	4.2	934				
0-19	2019	4.6	923		18.3		42.0
	2020	5.0	827	0.018	(3.4 - 30.9)	0.068	(-4.1 - 67.7)
	2021	3.1	616				
	2022	2.5	600				
	2018	5.0	580				
20-39	2019	7.0	699		10.6		9.5
	2020	6.9	634	0.125	(-3.1 - 22.5)	0.678	(-45.0 - 43.5)
	2021	4.7	548				
	2022	5.5	507				
	2018	6.3	1,630				
40-59	2019	7.9	1,788		12.2		26.1
	2020	7.3	1,622	0.002	(4.5 - 19.3)	0.037	(1.8 - 44.3)
	2021	6.4	1,385				
	2022	5.3	1,345				
	2018	7.0	6,193				
60-79	2019	7.0	6,615		11.6		32.1
	2020	6.6	6,500	0.000	(7.6 - 15.5)	0.000	(21.2 - 41.5)
	2021	5.7	6,168				
	2022	4.7	5,479				
	2018	7.4	4,001				
≥80	2019	7.7	4,112		12.6		28.0
	2020	6.6	4,268	0.000	(7.8 - 17.2)	0.000	(14.8 - 39.2)
	2021	5.2	4,168				
	2022	5.4	3,929				
	2018	6.7	13,338				
All	2019	7.2	14,137		12.1		28.7
	2020	6.6	13,851	0.000	(9.3 - 14.7)	0.000	(21.2 - 35.5)
	2021	5.5	12,885				
	2022	4.9	11,860				
Total number of *E. coli* isolates tested (n):					66,071		

Urine *E. coli*, FEMALE

Age group	Year	Observed resistance rate		2019-2022		2018/19 vs. 2022	
		ESBL+ (%)	Isolates tested (n)	p	AAD, % (95% CI)	p	Relative decrease, % (95% CI)
	2018	2.3	9,202				
0-19	2019	2.7	9,378		11.2		20.7
	2020	2.1	8,547	0.001	(4.9 - 17.1)	0.036	(1.5 - 36.2)
	2021	1.8	7,079				
	2022	2.0	6,189				
	2018	3.5	12,578				
20-39	2019	3.6	13,712		7.9		18.8
	2020	3.6	11,563	0.000	(3.6 - 12.1)	0.007	(5.6 - 30.2)
	2021	3.0	9,262				
	2022	2.9	8,726				
	2018	2.8	11,641				
40-59	2019	3.3	12,875		7.3		8.9
	2020	2.9	10,704	0.003	(2.5 - 11.8)	0.256	(-7.0 - 22.4)
	2021	2.5	8,628				
	2022	2.8	8,467				
	2018	2.6	39,844				
60-79	2019	3.0	38,780		13.5		34.5
	2020	2.8	34,895	0.000	(10.9 - 16.1)	0.000	(27.6 - 40.8)
	2021	2.5	32,015				
	2022	1.8	30,501				
	2018	3.0	38,712				
≥80	2019	3.1	36,721		12.4		33.3
	2020	3.0	35,589	0.000	(9.8 - 14.9)	0.000	(26.6 - 39.4)
	2021	2.6	32,174				
	2022	2.0	30,380				
	2018	2.9	111,977				
All	2019	3.1	111,466		11.7		28.7
	2020	2.9	101,298	0.000	(10.1 - 13.2)	0.000	(24.6 - 32.7)
	2021	2.5	89,158				
	2022	2.1	84,263				
Total number of *E. coli* isolates tested (n):					498,162		

Urine *E. coli*, BOTH SEXES

Age group	Year	Observed resistance rate		2019-2022		2018/19 vs. 2022	
		ESBL+ (%)	Isolates tested (n)	p	AAD, % (95% CI)	p	Relative decrease, % (95% CI)
	2018	2.5	10,136				
0-19	2019	2.8	10,301		12.4		24.2
	2020	2.4	9,374	0.000	(6.6 - 17.8)	0.007	(7.1 - 38.1)
	2021	1.9	7,695				
	2022	2.0	6,789				
	2018	3.5	13,158				
20-39	2019	3.8	14,411		8.0		17.5
	2020	3.8	12,197	0.000	(3.9 - 11.9)	0.009	(4.7 - 28.6)
	2021	3.1	9,810				
	2022	3.0	9,233				
	2018	3.2	13,271				
40-59	2019	3.9	14,663		8.0		12.1
	2020	3.5	12,326	0.000	(3.9 - 11.9)	0.072	(-1.1 - 23.6)
	2021	3.1	10,013				
	2022	3.1	9,812				
	2018	3.2	46,037				
60-79	2019	3.5	45,395		12.6		32.8
	2020	3.4	41,395	0.000	(10.4 - 14.8)	0.000	(26.9 - 38.2)
	2021	3.0	38,183				
	2022	2.3	35,980				
	2018	3.5	42,713				
≥80	2019	3.6	40,833		11.9		30.5
	2020	3.4	39,857	0.000	(9.7 - 14.2)	0.000	(24.4 - 36.1)
	2021	2.9	36,342				
	2022	2.4	34,309				
	2018	3.3	125,315				
All	2019	3.6	125,603		11.3		27.6
	2020	3.4	115,149	0.000	(10.0 - 12.7)	0.000	(23.9 - 31.1)
	2021	2.9	102,043				
	2022	2.5	96,123				
Total number of *E. coli* isolates tested (n):					564,233		

Blood *E. coli*, MALE

Age group	Year	Observed resistance rate		2019-2022		2018/19 vs. 2022	
		ESBL+ (%)	Isolates tested (n)	p	AAD, % (95% CI)	p	Relative decrease, % (95% CI)
	2018	13.6	22				
	2019	4.0	25		-11.6558		20.7
0-19	2020	6.1	33	0.765	(-130.3 - 45.9)	0.809	(-420.7 - 87.9)
	2021	3.3	30				
	2022	6.9	29				
	2018	14.8	27				
	2019	8.1	37		7.0		28.9
20-39	2020	7.0	43	0.778	(-54.1 - 43.9)	0.663	(-230.0 - 84.7)
	2021	5.9	34				
	2022	6.7	45				
	2018	10.3	213				
	2019	10.0	201		4.1		17.9
40-59	2020	10.9	202	0.662	(-15.8 - 20.6)	0.542	(-54.5 - 56.3)
	2021	11.0	228				
	2022	8.3	180				
	2018	9.4	882				
60-79	2019	8.1	1,025		10.5		33.6
	2020	8.1	1,019	0.026	(1.3 - 18.9)	0.012	(8.6 - 51.8)
	2021	7.0	1,067				
	2022	5.8	1,038				
	2018	9.3	611				
	2019	10.1	692		15.4		35.2
≥80	2020	7.6	727	0.005	(4.8 - 24.8)	0.023	(5.7 - 55.5)
	2021	6.6	760				
	2022	6.3	670				
	2018	9.6	1,755				
	2019	8.9	1,980		11.3		32.9
All	2020	8.2	2,024	0.001	(4.9 - 17.2)	0.000	(16.1 - 46.3)
	2021	7.2	2,119				
	2022	6.2	1,962				
Total number of *E. coli* isolates tested (n):					9,840		

Blood *E. coli*, FEMALE

Age group	Year	Observed resistance rate		2019-2022		2018/19 vs. 2022	
		ESBL+ (%)	Isolates tested (n)	p	AAD, % (95% CI)	p	Relative decrease, % (95% CI)
	2018	2.9	34				
0-19	2019	4.8	42		15.7		9.5
	2020	6.8	44	0.581	(-54.8 - 54.1)	0.943	(-1296.8 - 94.1)
	2021	5.9	34				
	2022	2.4	42				
	2018	7.1	126				
20-39	2019	5.7	158		-20.2		-59.1036
	2020	6.7	164	0.172	(-56.7 - 7.7)	0.272	(-264.6 - 30.6)
	2021	7.4	148				
	2022	10.1	119				
	2018	4.4	272				
40-59	2019	7.1	309		18.0		30.2
	2020	5.3	281	0.082	(-2.5 - 34.4)	0.340	(-46.2 - 66.7)
	2021	4.3	299				
	2022	4.1	269				
	2018	6.5	1,112				
60-79	2019	6.8	1,176		16.4		37.0
	2020	4.7	1,055	0.003	(6.0 - 25.7)	0.014	(9.1 - 56.4)
	2021	4.1	1,107				
	2022	4.2	1,004				
	2018	5.8	1,224				
≥80	2019	6.2	1,252		11.0		24.8
	2020	6.2	1,249	0.030	(1.1 - 19.9)	0.104	(6.0 - 46.7)
	2021	4.8	1,321				
	2022	4.5	1,179				
	2018	6.0	2,768				
All	2019	6.5	2,937		12.0		26.6
	2020	5.6	2,793	0.000	(5.6 - 18.0)	0.007	(8.0 - 41.5)
	2021	4.6	2,909				
	2022	4.6	2,613				
Total number of *E. coli* isolates tested (n):					14,020		

Blood *E. coli*, BOTH SEXES

Age group	Year	Observed resistance rate		2019-2022		2018/19 vs. 2022	
		ESBL+ (%)	Isolates tested (n)	p	AAD, % (95% CI)	p	Relative decrease, % (95% CI)
	2018	7.1	56				
0-19	2019	4.5	67		5.4		14.1
	2020	6.5	77	0.811	(-49.7 - 40.3)	0.849	(-310.2 - 82.0)
	2021	4.7	64				
	2022	4.2	71				
	2018	8.5	153				
20-39	2019	6.2	195		-13.5		-32.6
	2020	6.8	207	0.287	(-43.4 - 10.1)	0.448	(-174.8 - 36.0)
	2021	7.1	182				
	2022	9.1	164				
	2018	7.0	485				
40-59	2019	8.2	510		10.0		24.3
	2020	7.7	483	0.149	(-3.9 - 22.1)	0.259	(-22.7 - 53.2)
	2021	7.2	527				
	2022	5.8	449				
	2018	7.8	1,994				
60-79	2019	7.4	2,201		12.6		34.1
	2020	6.4	2,074	0.000	(5.8 - 18.9)	0.001	(16.2 - 48.2)
	2021	5.5	2,174				
	2022	5.0	2,042				
	2018	7.0	1,835				
≥80	2019	7.6	1,944		12.9		29.2
	2020	6.7	1,976	0.001	(5.8 - 19.5)	0.008	(8.7 - 45.0)
	2021	5.5	2,081				
	2022	5.1	1,849				
	2018	7.4	4,523				
All	2019	7.5	4,917		11.4		29.0
	2020	6.7	4,817	0.000	(6.9 - 15.6)	0.000	(16.7 - 39.4)
	2021	5.7	5,028				
	2022	5.3	4,575				
Total number of *E. coli* isolates tested (n):					23,860		
