# Supplementary material for: Impact of the COVID-19 pandemic on extended-spectrum β-lactamase producing Escherichia coli in urinary tract and blood stream infections: results from a nationwide surveillance network, Finland, 2018 to 2022
Source: Antimicrob Resist Infect Control. 2024 Jul 6;13:72. doi: 10.1186/s13756-024-01427-z (PMC11227720; doi:10.1186/s13756-024-01427-z)
Supplement: Supplementary file 2 — Supplementary Material 2 [file 13756_2024_1427_MOESM2_ESM.pdf]

Supplementary Table S2:

Supplementary Table S2: The annual incidence of ESBL-producing *Escherichia coli* in urine and blood cultures and their estimated annual decreaces stratified by age groups and both sexes, Finland, 2018-2022. AAD, Average annual decreace; ESBL+, extended-spectrum β-lactamase-producing *E. coli*; CI, combatibility interval.

Urine ESBL+ *E. coli*, MALE							
		Observed insidence rate ratio (IRR)		2019-2022		2018/2019 vs. 2022	
Age group	Year	Incidence / 100,000 inhabitants	ESBL+ *E. coli* isolates (n)	p	AAD, % (95% CI)	p	Relative decrease, % (95% CI)
0-19	2018	7.0	39				
	2019	7.6	42		30.0		62.0
	2020	7.5	41	0.000	(17.0-41.0)	0.001	(31.3 - 79.0)
	2021	3.5	19				
	2022	2.8	15				
20-39	2018	4.5	29				
	2019	7.6	49		20.1		28.2
	2020	6.8	44	0.003	(7.3 - 31.0)	0.18	(-16.6 - 55.8)
	2021	4.0	26				
	2022	4.3	28				
40-59	2018	15.9	102				
	2019	22.3	142		20.8		41.4
	2020	18.7	119	0.035	(13.5 - 27.4)	0.000	(21.5 - 56.3)
	2021	14.0	89				
	2022	11.2	71				
60-79	2018	80.7	432				
	2019	85.0	460		17.6		44.1
	2020	77.7	426	0.000	(13.7-21.3)	0.000	(34.9 - 52.0)
	2021	63.6	354				
	2022	46.3	259				
≥80	2018	317.7	296				
	2019	327.0	318		17.0		39.7
	2020	279.1	283	0.000	(12.3 - 21.5)	0.000	(28.2 - 49.4)
	2021	209.0	216				
	2022	194.3	214				
All	2018	36.4	898				
	2019	40.9	1,011		17.2		39.0
	2020	36.9	913	0.000	(14.5 - 19.7)	0.000	(32.3 - 44.9)
	2021	28.4	704				
	2022	23.6	587				
Total number of ESBL+ *E. coli* isolates (n):					4,113		

Urine ESBL+ *E. coli*, FEMALE							
		Observed insidence rate ratio (IRR)		2019-2022		2018/2019 vs. 2022	
Age group	Year	Incidence / 100,000 inhabitants	ESBL+ *E. coli* isolates (n)	p	AAD, % (95% CI)	p	Relative decrease, % (95% CI)
0-19	2018	40.5	214				
	2019	47.5	249		22.4		46.1
	2020	35.2	183	0.000	(16.8 - 27.5)	0.000	(32.9 - 56.7)
	2021	25.0	129				
	2022	23.7	122				
20-39	2018	71.4	435				
	2019	81.4	494		21.6		45.8
	2020	68.3	414	0.000	(17.8 - 25.2)	0.000	(36.8 - 53.6)
	2021	46.3	280				
	2022	41.4	250				
40-59	2018	51.2	321				
	2019	68.8	429		20.1		36.1
	2020	50.6	314	0.000	(15.9 - 24.2)	0.000	(24.7 - 45.7)
	2021	35.1	217				
	2022	38.4	236				
60-79	2018	176.0	1,052				
	2019	190.3	1,148		21.2		50.8
	2020	159.1	969	0.000	(18.8 - 23.6)	0.000	(45.5 - 55.6)
	2021	127.3	787				
	2022	90.1	559				
≥80	2018	686.3	1,180				
	2019	646.6	1,133		19.7		50.4
	2020	602.7	1,080	0.000	(17.3 - 22.1)	0.000	(45.3 - 55.0)
	2021	454.4	821				
	2022	330.6	621				
All	2018	126.4	3,202				
	2019	136.3	3,453		20.0		46.4
	2020	116.8	2,960	0.000	(18.6 - 21.4)	0.000	(43.3 - 49.4)
	2021	88.0	2,234				
	2022	70.3	1,788				
Total number of ESBL+ *E. coli* isolates (n):					13,637		

Urine ESBL+ *E. coli*, BOTH SEXES							
		Observed resistance rate		2019-2022		2018/2019 vs. 2022	
Age group	Year	Incidence / 100,000 inhabitants	ESBL+ *E. coli* isolates (n)	p	AAD, % (95% CI)	p	Relative decrease, % (95% CI)
0-19	2018	23.4	253				
	2019	27.1	291		23.5		48.4
	2020	21.0	224	0.000	(18.4 - 28.2)	0.000	(36.6 - 58)
	2021	14.0	148				
	2022	13.0	137				
20-39	2018	36.9	464				
	2019	43.3	543		21.5		44.6
	2020	36.6	458	0.000	(17.9 - 25.0)	0.000	(35.9 - 52.2)
	2021	24.5	306				
	2022	22.2	278				
40-59	2018	33.4	423				
	2019	45.3	571		20.4		37.5
	2020	34.5	433	0.000	(16.7 - 23.8)	0.000	(28.0 - 45.8)
	2021	24.4	306				
	2022	24.6	307				
60-79	2018	131.0	1,484				
	2019	140.5	1,608		20.2		48.9
	2020	120.5	1,395	0.000	(18.1 - 22.2)	0.000	(44.4 - 53.1)
	2021	97.1	1,141				
	2022	69.3	818				
≥80	2018	556.8	1,476				
	2019	532.6	1,451		19.3		48.5
	2020	485.7	1,363	0.000	(17.2 - 21.4)	0.000	(44.0 - 52.7)
	2021	365.1	1,037				
	2022	280.2	835				
All	2018	82.0	4,100				
	2019	89.2	4,464		19.4		44.8
	2020	77.3	3,873	0.000	(18.1 - 20.6)	0.000	(42.0 - 47.5)
	2021	58.5	2,938				
	2022	47.2	2,375				
Total number of ESBL+ *E. coli* isolates (n):					17,750		

Blood ESBL+ *E. coli*, MALE							
		Observed insidence rate ratio (IRR)		2019-2022		2018/2019 vs. 2022	
Age group	Year	Incidence / 100,000 inhabitants	ESBL+ *E. coli* isolates (n)	p	AAD, % (95% CI)	p	Relative decrease, % (95% CI)
0-19	2018	0.54	3				
	2019	0.18	1		-15.2		-2.6
	2020	0.37	2	0.701	(-136.8 - 44.0)	0.980	(-628.3 - 85.5)
	2021	0.19	1				
	2022	0.37	2				
20-39	2018	0.62	4				
	2019	0.46	3		3.6		14.3
	2020	0.46	3	0.891	(-63.6 - 43.2)	0.844	(-300.5 - 81.7)
	2021	0.31	2				
	2022	0.46	3				
40-59	2018	3.4	22				
	2019	3.1	20		5.5		28.1
	2020	3.5	22	0.565	(-14.7 - 22.2)	0.328	(-39.4 - 63.0)
	2021	3.9	25				
	2022	2.4	15				
60-79	2018	15.5	83				
	2019	15.3	83		10.8		30.4
	2020	15.1	83	0.028	(1.3 - 19.4)	0.032	(3.0 - 50.1)
	2021	13.5	75				
	2022	10.7	60				
≥80	2018	61.2	57				
	2019	72.0	70		18.4		42.8
	2020	54.2	55	0.001	(8.0 - 27.7)	0.005	(15.6 - 61.3)
	2021	48.4	50				
	2022	38.1	42				
All	2018	6.8	169				
	2019	7.2	177		11.1		30.0
	2020	6.7	165	0.001	(4.5 - 17.2)	0.003	(11.7 - 44.5)
	2021	6.2	153				
	2022	4.9	122				
Total number of ESBL+ *E. coli* isolates (n):					786		

Blood ESBL+ *E. coli*, FEMALE							
		Observed insidence rate ratio (IRR)		2019-2022		2018/2019 vs. 2022	
Age group	Year	Incidence / 100,000 inhabitants	ESBL+ *E. coli* isolates (n)	p	AAD, % (95% CI)	p	Relative decrease, % (95% CI)
0-19	2018	0.19	1				
	2019	0.38	2		17.8		31.8
	2020	0.58	3	0.543	(-54.4 - 56.2)	0.767	(-756.5 - 94.6)
	2021	0.39	2				
	2022	0.19	1				
20-39	2018	1.5	9				
	2019	1.5	9		-8.9		-34.2
	2020	1.8	11	0.532	(-42.4 - 16.7)	0.505	(-218.4 - 43.5)
	2021	1.8	11				
	2022	2.0	12				
40-59	2018	1.9	12				
	2019	3.5	22		20.4		34.3
	2020	2.4	15	0.051	(-0.1 - 36.7)	0.278	(-40.4 - 69.2)
	2021	2.1	13				
	2022	1.8	11				
60-79	2018	12.0	72				
	2019	13.3	80		20.7		46.5
	2020	8.2	50	0.000	(10.5 - 29.8)	0.001	(2.0 - 63.3)
	2021	7.3	45				
	2022	6.8	42				
≥80	2018	41.3	71				
	2019	43.9	77		13.7		33.8
	2020	43.0	77	0.007	(3.9 - 22.5)	0.022	(5.8 - 53.5)
	2021	35.4	64				
	2022	28.2	53				
All	2018	6.5	165				
	2019	7.5	190		14.6		33.2
	2020	6.2	156	0.000	(8.2 - 20.6)	0.001	(15.7 - 47.0)
	2021	5.3	135				
	2022	4.7	119				
Total number of ESBL+ *E. coli* isolates (n):					765		

Blood ESBL+ *E. coli*, BOTH SEXES							
		Observed resistance rate		2019-2022		2018/2019 vs. 2022	
Age group	Year	Incidence / 100,000 inhabitants	ESBL+ *E. coli* isolates (n)	p	AAD, % (95% CI)	p	Relative decrease, % (95% CI)
0-19	2018	0.37	4				
	2019	0.28	3		4.9		12.2
	2020	0.47	5	0.834	(-52.0 - 40.5)	0.869	(-310.5 - 81.2)
	2021	0.28	3				
	2022	0.28	3				
20-39	2018	1.0	13				
	2019	1.0	12		-6.2		-20.3
	2020	1.1	14	0.623	(-34.8 - 16.4)	0.629	(154.9 - 43.2)
	2021	1.0	13				
	2022	1.2	15				
40-59	2018	2.7	34				
	2019	3.3	42		12.1		30.8
	2020	2.9	37	0.087	(-1.9 - 24.2)	0.148	(-13.9 - 58.0)
	2021	3.0	38				
	2022	2.1	26				
60-79	2018	13.7	155				
	2019	14.2	163		15.0		38.1
	2020	11.5	133	0.000	(8.2 - 21.4)	0.000	(20.6 - 51.7)
	2021	10.2	120				
	2022	8.6	102				
≥80	2018	48.3	128				
	2019	54.0	147		15.7		37.7
	2020	47.0	132	0.000	(8.7 - 22.2)	0.000	(19.1 - 52.0)
	2021	40.1	114				
	2022	31.9	95				
All	2018	6.7	334				
	2019	7.3	367		12.8		31.6
	2020	6.4	321	0.000	(8.3 - 17.1)	0.000	(19.4 - 42.0)
	2021	5.7	288				
	2022	4.8	241				
Total number of ESBL+ *E. coli* isolates (n):					1,551		
